# Effect of long-term in-row branch covering on soil microorganisms in pear orchards

**DOI:** 10.1515/biol-2022-0807

**Published:** 2024-01-29

**Authors:** Minghui Ji, Jintao Xu, Lijuan Gao, Longfei Li, Huan Liu, Baofeng Hao

**Affiliations:** Changli Institute of Pomology HAAFS, Qinhuangdao, Hebei, 066600, China

**Keywords:** in-row coverage mode, soil properties, microbial diversity, vertical distribution

## Abstract

Branches covering (BC) is a way to reuse the pruned branches and save the cost of ground cloth. This study investigated the effects of BC and ground-cloth covering on the soil microcosm environment by measuring the chemical properties and microbial communities at different soil depths for 6 years. The results revealed that BC significantly improved soil chemical properties, increased the abundance of bacterial microbial communities and the diversity and homogeneity of bacteria and fungi, while decreased the abundance of fungal microbial communities. There was a threshold value for the regulation of microbial communities by BC, which decreased the high-abundance communities (*Proteobacteria*, *Ascomycota*, etc.) and increased the low-abundance communities (*Acidobacteriota*, *Basidiomycota*, etc.). Fungi were more sensitive to BC than bacteria. The stability and homogeneity of microorganisms were stronger in the 15–25 cm soil layer. The bacterial phyla were dominated by *Proteobacteria*, with the top 10 phyla accounting for more than 80% of the relative abundance; the genera were dominated by *MND1*, with the top 10 genera accounting for about 10%. The fungal phyla were dominated by *Ascomycota*, with the top 10 phyla accounting for 50–90%; the genera were dominated by *unidentified Pyronemataceae* sp., with the top 10 genera accounting for 30–60%. The phyla that differed significantly between treatments were mainly *Proteobacteria*, *Ascomycota*, *Acidobacteriota*, and *Basidiomycota*. In addition, metabolism was the predominant function in bacteria, while Saprotroph was the predominant function in fungi. Bacteroidota correlated strongly with soil chemical properties and bacterial functions, while Chytridiomycota correlated strongly with soil chemical properties and Pathogen-Saprotroph-Symbiotroph. In conclusion, BC can improve soil nutrient content and optimize microbial community structure and function. Through initially assessing the effects of BC on soil nutrients and microorganisms in pear orchard rows, this study provides a reference for excavating key microorganisms and updating the soil row management model.

## 1 Introduction

Soil microorganism has a strong relation with the soil microenvironment, and there is a significant relationship between microbial functional diversity and species [1,2]. Protists stimulate plant growth by interacting with microbiota [3]. Soil types, such as forest land and agricultural land, mediate soil apoplastic decomposition by influencing the microbial community succession [4]. The uncertainty of soil organic matter’s (SOM) composition and origin is uncertain as a soil carbon reservoir interferes with soil sustainable development [5]. Increasing evidence indicates that microorganism affects the transformation and degradation of SOM [6]. Microbial rates and microbial community diversity are strongly dependent on climate [7,8]. Research has shown that biochar regulates bacterial abundance by influencing soil properties and microbial carbon efficiency is positively correlated with bacterial diversity, while fungal diversity is influenced by biochar’s concentration and cracking temperature [9,10]. Fungal abundance can also affect the relation between biochar and bacterial abundance. By controlling biochar’s density and production conditions, it is possible to adjust soil microbial function and promote nitrogen-fixing capacity [11]. Real-time changes in microbial community activity suggest that dissolved organic carbon and particulate organic carbon affect fungal communities, while bacterial communities are affected by microbial biomass carbon regardless of fertilization method [12]. Some microbes, such as *Streptomyces*, affect microbial communities by producing metabolites [13]. The rhizosphere, as a plant–microbe–soil interface, has significant differences in microbial communities and diverse functions [14]. Conventional rhizosphere fungi have relatively high species richness and are generally enriched in *Glomeromycetes* [15]. Rhizosphere microorganisms reduce embolization of the endodermis and improve plant stress tolerance by decreasing transcription of ABA [16]. Root exudates promote the assembly process of plant rhizosphere microbiota [17].

Cultivation techniques have a significant influence on soil microorganisms. Agronomic management methods have altered the interaction between plants and rhizosphere microorganisms at both the taxonomic and functional genomic levels. For instance, long-term nitrogen application increases the microbial abundance in nitrogen recycle, reduces the microbial abundance in nitrogen fixation, and affects the balance of the soil ecosystem [18,19]. Continuous cropping can lead to secondary salinization of soil, loss of microbial diversity, and accumulation of pathogenic fungi [20]. Both replantation and planting year significantly affect bacterial and fungal community composition [21,22].

Researchers have found several solutions to solve soil microecology issues. For example, increasing plant diversity can increase soil microbial diversity and reduce the spread of soil-borne pathogens [23]. Grasses improve rhizosphere symbiosis environment, microbial populations, and topsoil rhizosphere enzyme activities [24,25]. Crop rotation systems improve SOM storage by increasing carbon input [26]. Cover crops have been shown to enrich bacterial communities according to soil types; in clay soils, the fixation of microbial biomass P is promoted, while in sandy soils, the acquisition of microbial biomass C and N is enhanced [27,28,29]. In addition, the application of organic fertilizer significantly increased the biomass and diversity of soil microorganisms and improved the activity of soil-borne microorganisms [30]. Soil inoculation with mixed inoculants promotes plant growth and nutrient uptake, but transplanting will lead to ineffectiveness [31]. W19 (a biocontrol bacteria) can inhibit pathogen growth in the rhizosphere, negatively affect pathogen population density, and activate related microorganisms that benefit plants [32].

**Figure 1:**
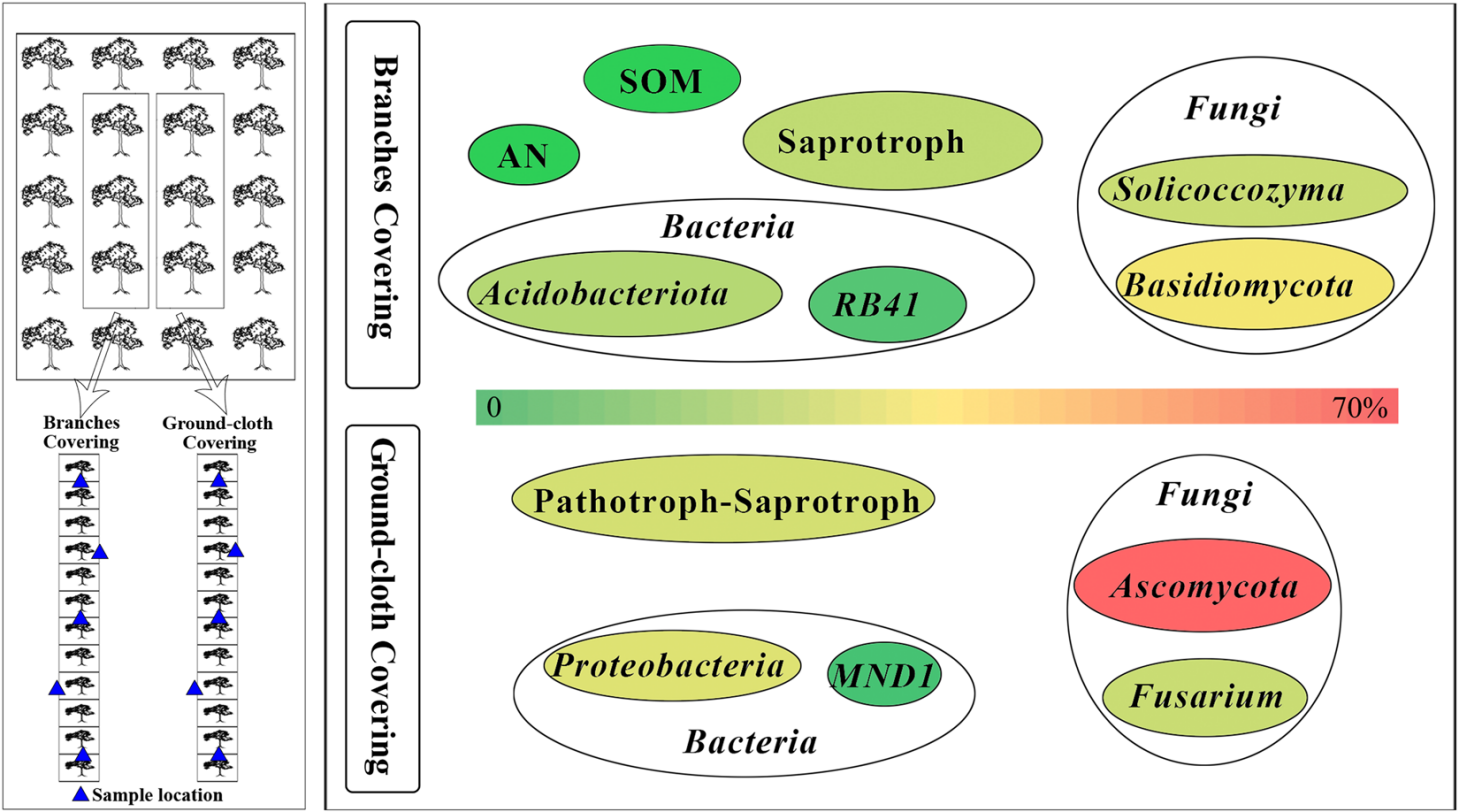
Design and results schematic of experiment (the left side of the figure shows the experimental treatments and the right side shows the main results. BC: soil in the row covered with crushed branches; GC: soil in the row covered with black ground-cloth).

Currently, there is limited research on the effect of covering on microbes under soil vertical distribution, although the predictability of microbial communities increases with the observed soil depth [33]. In addition, fruit trees produce many branches due to annual pruning, which are difficult to manage. Studying the effects of branches covering (BC) on soil microorganisms at different soil depths can help analyze the impact of BC on the soil microecological environment and explore the feasibility of using BC to solve these problems. Starting in 2015, pruned branches were crushed using a branch crusher and covered into the rows as a treatment, with ground-cloth covering (GC) as a control. In 2021, the effects of long-term BC on soil and soil microorganisms were analyzed by measuring soil chemical properties and sequencing the microorganisms at different depths under both modes, aiming to clarify the community structure of bacteria and fungi under BC (Figure 1).

## 2 Materials and methods

### 2.1 Experimental design and sampling

**Figure 2:**
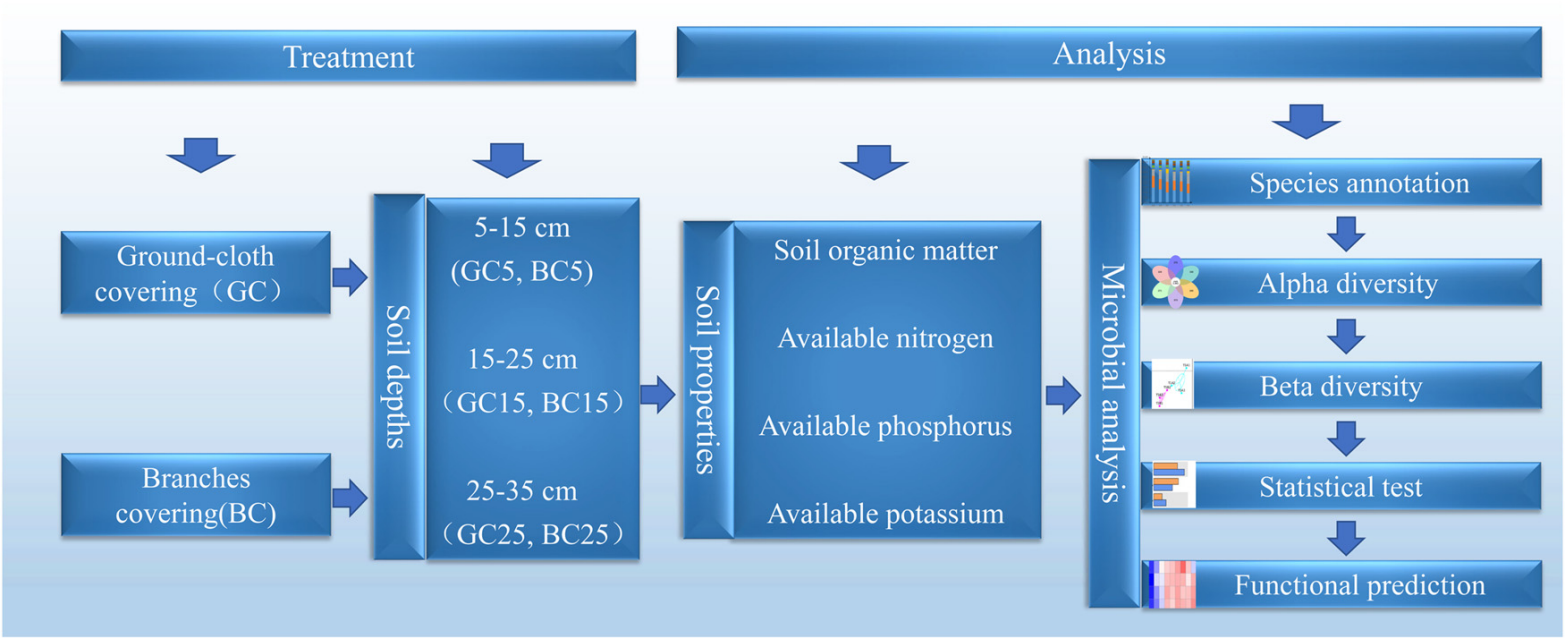
Experimental design diagram.

Soil samples were collected in April 2021 from a pear orchard at Kongzhuang experimental station of Changli Institute of Pomology HAAFS (39°42′N, 119°5′E), with GC and BC from 2015 to 2021. The soil type was clay. Soil moisture content was 22% and pH = 6.9 for GC, and soil moisture content was 27% and pH = 7.1 for BC. To accurately represent the soil near the root system of pear trees, samples were taken 50 cm from the main trunk. Five portions of soil at three different depths of 5 (5–15 cm), 15 (15–25 cm), and 25 (25–35 cm) were taken in both GC and BC, respectively, which were recorded as GC5, GC15, GC25, BC5, BC15, and BC25. Soil samples collected at different depths were mixed separately and retained. The samples were divided into two parts; one for soil chemical property determination and the other was sent to Novogene (Beijing, China) for sequencing analysis of bacteria and fungi using a PE250 sequencing system (Figure 2).

### 2.2 Soil indexes determination

SOM was determined using the potassium dichromate volumetric method (external heating method); Soil available nitrogen (AN) was determined using the alkaline hydrolysis diffusion method; soil available phosphorus (AP) was determined using the antimony anti-colorimetric method [34]. Soil available potassium (AK) was determined using the ammonium acetate extraction method [35].

### 2.3 DNA sequencing and analysis

Total genome DNA from soil samples was extracted using the CTAB method. The amplified regions were 16sV3-V4 and ITS1-5F. The specific primers of 16S were 341F (CCTAYGGGRBG-CASCAG) and 806R (GGACTACNNGGGTATCTAAT), and the ITS amplicon sequencing used the specific primers ITS5-1737F (GGAAGTAAAAGTCGTAACAAGG) and ITS2-2043R (GCTGCGTTCTTCATCGATGC). The library was constructed using TruSeq^®^ DNA PCR-Free Sample Preparation Kit and sequenced.

The downstream data were split according to the Barcode sequence and PCR products and then were spliced using FLASH (V1.2.7, http://ccb.jhu.edu/software/FLASH/) to obtain Raw Tags. The Clean Tags were obtained by filtering with Qiime (V1.9.1, http://qiime.org/scripts/split_libraries_fastq.html). Chimeras were removed to obtain Effective Tags by comparing with the species annotation database (https://github.com/torognes/vsearch/). Using the Uparse algorithm (Uparse v7.0.1001, http://www.drive5.com/uparse/), the Effective Tags were clustered into Operational Taxonomic Units (OTUs) with 97% identity and species were annotated. Among them, the Mothur method with SILVA138 (http://www.arb-silva.de/) for SSUrRNA database (threshold: 0.8–1) was selected for 16S; blast method (http://qiime.org/scripts/assign_taxonomy.html) in Qiime software (Version 1.9.1) and the Unit (v8.2) database (https://unite.ut.ee/) was selected for ITS. Sequence comparison was subsequently performed using MUSCLE (Version 3.8.31, http://www.drive5.com/muscle/) software. Data were normalized based on the least amount of data in the samples. Among them, Alpha Diversity and Beta Diversity were performed using Qiime software (Version 1.9.1), and bacterial and fungal function prediction was performed according to Tax4Fun and FunGuild, respectively. Tukey test, non-metric multidimensional scaling (NMDS), and PCA analysis were performed using R software (Version 2.15.3). Amplicon data analysis and plotting were completed on the Novogene Tools platform (https://magic.novogene.com/customer/main#).

Data were processed using Office 365 and analyzed using analysis of variance (ANOVA) with SPSS 20.0. The correlation between soil chemical properties and microbial community was analyzed using the Pearson correlation coefficient method. The interaction between cover pattern and soil depth had minimal effect on dominant species and microbial functions, so only a two-factor ANOVA was conducted on soil chemical properties.

## 3 Result

### 3.1 Chemical properties of soil

The content of SOM, AN, and AP was greater in BC than in GC, while the content of AK was greater in GC than in BC (Table 1, Coverage model). Soil chemical properties decreased significantly with soil depth (*P* < 0.001) (Table 1, Soil depth). As for the Coverage model and Coverage model × Soil depth in *F* valve were ranked as SOM > AN > AK > AP (Soil depth), Soil depth > Coverage model (AN, AP, AK), Soil depth < Coverage model (SOM). It indicates that depth has a stronger effect on AN, AP, and AK, and the coverage model has a more pronounced effect on SOM. The interaction between Coverage model and Soil depth was significant. For the same soil parameter, the *F* values of Coverage model × Soil depth were smaller than those for Coverage model and Soil depth. As soil depth increased or in the order of SOM, AN, AP, and AK, the effect of BC gradually decreased and negative values appeared in AP and AK. However, the effect on AP and AK was smaller (Table 1, BC/GC-1).

**Table 1:** Analysis of soil chemical properties

		SOM (%)	AN (mg/kg)	AP (mg/kg)	AK (mg/kg)
Coverage model	GC	0.81 ± 0.21b	85.83 ± 17.08b	121.40 ± 24.39b	362.64 ± 112.74a
	BC	1.39 ± 0.56a	120.21 ± 39.60a	130.94 ± 40.59a	347.63 ± 130.23b
	*F*	450.67	128.37	5.38	16.12
	*P*	***	***	*	***
Soil depth	5–15 cm	1.57 ± 0.55a	137.38 ± 34.32a	157.43 ± 13.35a	502.40 ± 10.72a
	15–25 cm	1.03 ± 0.30b	97.87 ± 18.16b	134.37 ± 16.47b	339.20 ±± 16.08b
	25–35 cm	0.70 ± 0.11c	73.82 ± 7.75c	86.72 ± 9.95c	223.82 ± 16.73c
	*F*	344.00	149.19	102.36	1869.42
	*P*	***	***	***	***
Coverage model × Soil depths	*F*	72.67	24.42	9.30	11.92
	*P*	***	***	***	***
BC/GC-1	BC5/GC5-1	0.96	0.58	0.13	0.02
	BC15/GC15-1	0.71	0.38	0.21	−0.08
	BC25/GC25-1	0.38	0.15	−0.16	−0.12

Note: *F*: *F*-value; *P*: significance; Coverage model × Soil depths: interaction of cover pattern and soil depth; BC/GC-1: indicates soil nutrient changes in BC relative to GC; the same below.

*: *p* < 0.05; **: *p* < 0.01; ***: *p* < 0.001.

### 3.2 Diversity of soil microbial community

**Figure 3:**
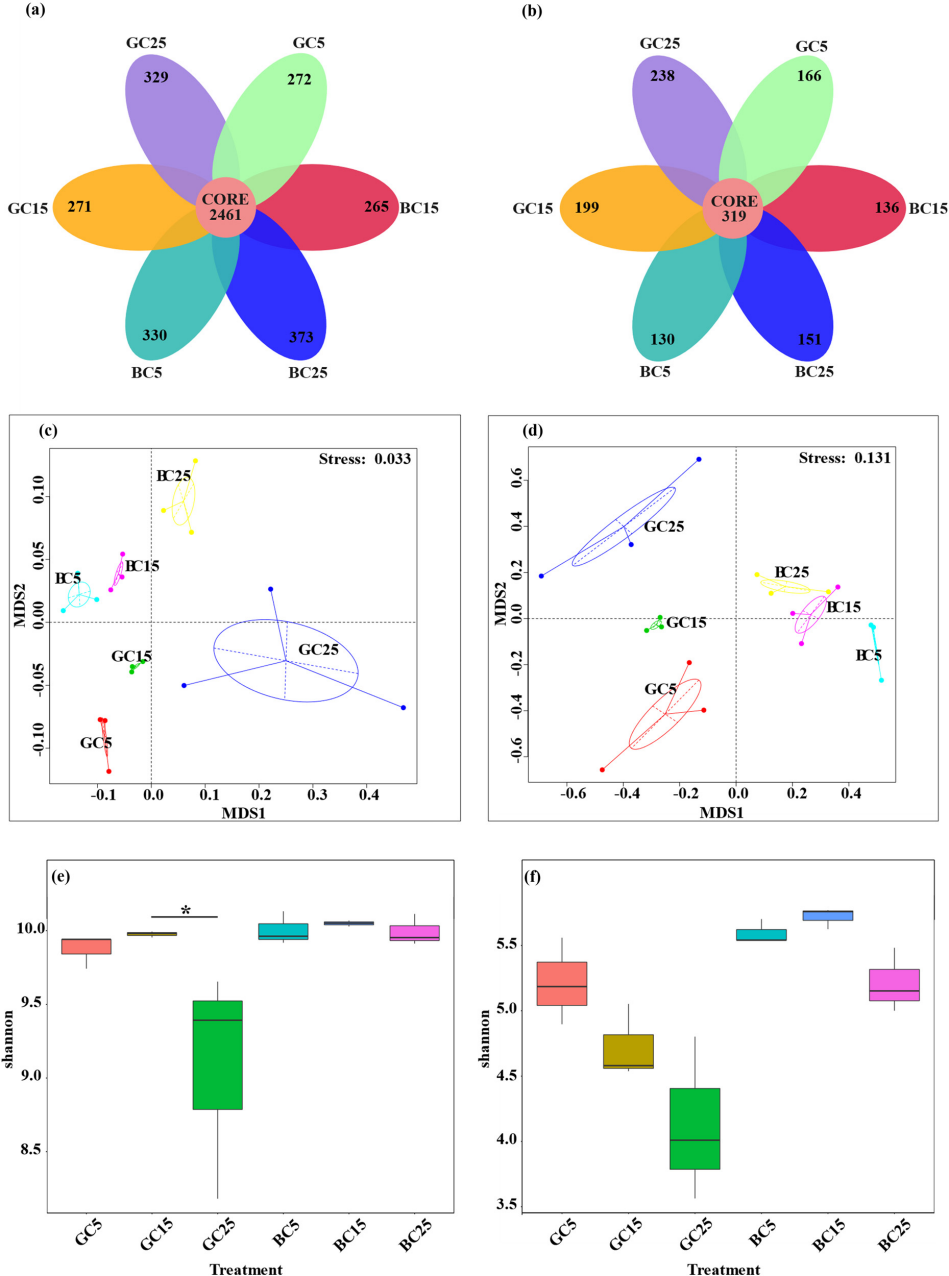
OTU analysis of bacterial and fungal communities in different covering methods (a and b). NMDS analysis of bacterial and fungal communities in different covering methods (c and d). Alpha diversity analysis of bacterial and fungal communities in different covering methods (e and f).

The bacterial microorganisms had 3333 OTUs in GC and 3429 OTUs in BC, with 2461 identical OTUs (Figure 3a). The fungal microorganisms had 922 OTUs in GC and 736 OTUs in BC, with 319 identical OTUs (Figure 3b). In each soil layer, the number of bacterial OTUs in BC was greater than in GC (with GC15 approximately equal to BC15), while the number of fungal OTUs was greater in GC than in BC. It indicates that the abundance of bacterial microorganisms was greater than that of fungi and that BC was beneficial for increasing bacterial microorganisms and decreasing fungal microorganisms. In bacteria, the unique OTUs were only 0.11–0.15 times the number of identical OYUs, while in fungi, the unique OTUs were 0.28–0.69 times the number of the identical OTUs. This indicates that bacteria are more stable and conserved while fungi are more sensitive to changes in the soil environment.

The MRPP (multiresponse permutation procedures) analysis showed that all the differences between groups were greater than the differences within groups. The NMDS plot showed that in the bacterial microbial community, the distance was GC15 < GC5 < GC25 and BC15 < BC5 < BC25; the density was GC5 > BC5, GC15 > BC15, and GC25 < BC25 (Figure 3c). In the fungal microbial community, the distance was GC15 < GC25 < GC5 and BC15 < BC25 < BC5; the density was: GC5 < BC5, GC15 > BC15, and GC25 < BC25 (Figure 3d). In both bacteria and fungi, the densities of GC and BC samples in each treatment were: 15 > 5 > 25 (with similar densities for BC in each soil layer for fungi). This indicates that the stability and homogeneity of microorganisms in the 15–25 cm soil samples were stronger than in the 5–15 and 25–35 cm soil samples and less influenced by BC.

The bacterial and fungal box plots showed that BC can increase the diversity of soil micro-organisms and improve their homogeneity. There was also a small variation difference between GC and BC in the 15–25 cm depth soil layer, indicating that this soil layer is more stable than other soil layers. This is consistent with the NMDS analysis. The difference in bacteria between GC25 and BC25 was significant.

### 3.3 Analysis of dominant soil microbial populations

**Figure 4:**
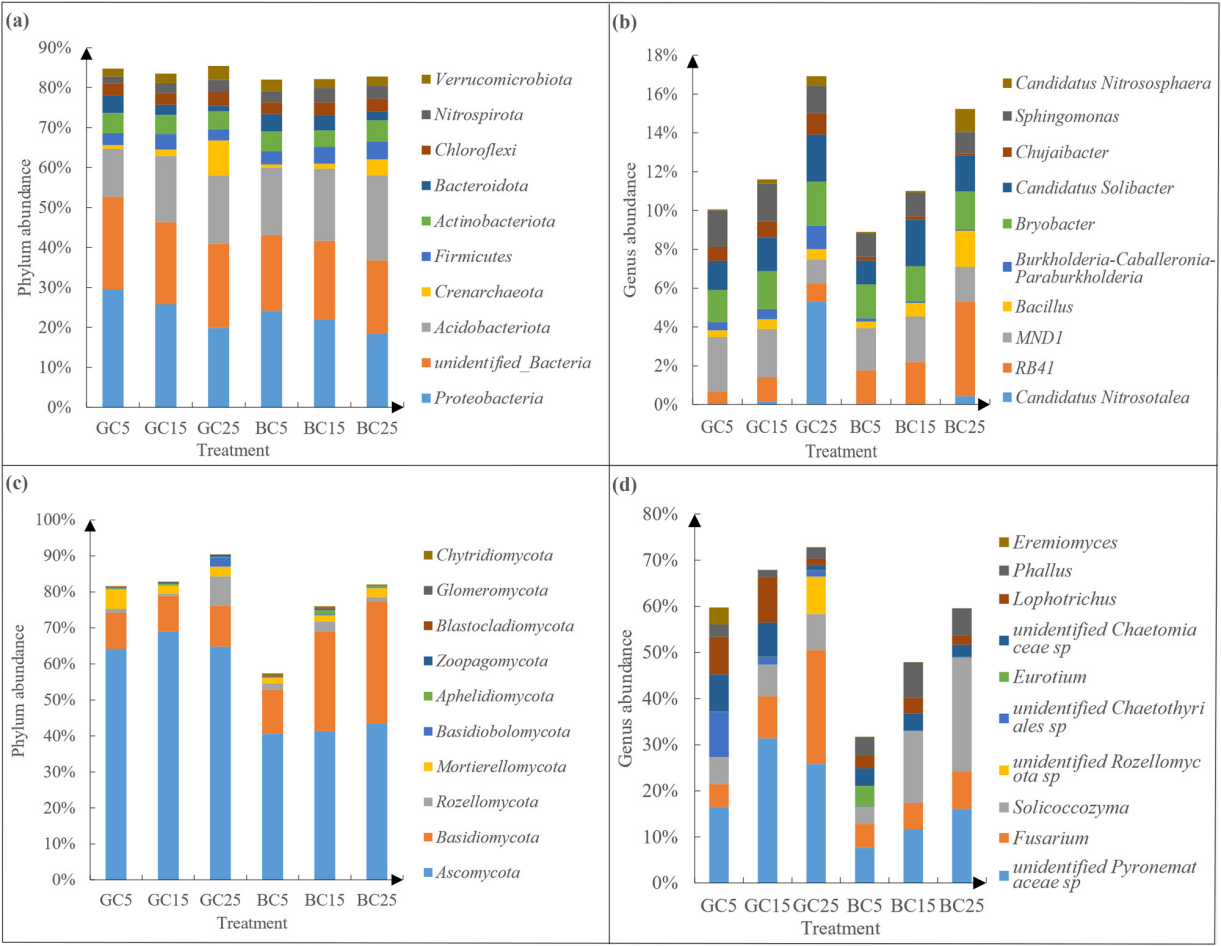
Top 10 phyla (a and c) and genera (b and d) of bacterial and fungal communities in different covering methods.

The bacterial phyla were dominated by *Proteobacteria* (GC5: 29.41%, GC15: 25.78%, GC25: 19.83%, BC5: 24.16%, BC15: 21.89%, BC25: 18.35%), *unidentified Bacteria* (GC5: 23.30%, GC15: 20.58%, GC25: 21.12%, BC5: 18.99%, BC15: 19.74%, BC25: 18.44%), and *Acidobacteriota* (GC5: 11.96%, GC15: 16.53%, GC25: 17.04%, BC5: 16.82%, BC15: 18.05%, BC25: 21.17%) (Figure 4a). As the soil layer deepened, the relative abundances of *Proteobacteria*, *unidentified Bacteria*, and *Bacteroidota* were decreased, while those of *Acidobacteriota* and *Crenarchaeota* were increased. In the same soil layer, the relative abundances of *Proteobacteria*, *unidentified Bacteria*, and *Crenarchaeota* were higher in GC than in BC. In contrast, the relative abundances of *Acidobacteriota*, *Firmicutes*, *Bacteroidota*, and *Nitrospirota* were lower in GC than in BC. The relative total abundance of the first 10 phyla was similar across treatments and all were greater than 80%.

*MND1* (GC5:2.79%, GC15:2.49%, GC25:1.23%, BC5:2.18%, BC15:2.35%, BC25:1.82%), *RB41* (GC5:0.65%, GC15:1.24%, GC25:0.96%, BC5:1.73%, BC15:2.18%, BC25:4.85%), *Bryobacter* (GC5:1.66%, GC15:1.95%, GC25:2.27%, BC5:1.75%, BC15:1.79%, BC25:1.94%), and *Candidatus Solibacter* (GC5:1.51%, GC15:1.74%, GC25:2.42%, BC5:1.23%, BC15:2.39%, BC25:1.86%) were predominant species in the bacterial genera (Figure 4b). With the soil depth increased, the amount of *Bacillus*, *Bryobacter*, and *Candidatus Nitrososphaera* increased; the abundance of *Burkholderia-Caballeronia-Paraburkholderia* and *Chujaibacter* increased gradually in GC while decreased in BC. In the same soil layer, the relative abundances of *Candidatus Nitrosotalea*, *Burkholderia-Caballeronia-Paraburkholderia*, *Chujaibacter*, and *Sphingomonas* were greater in GC than in BC, while that of *RB41* was less in GC than in BC. The total abundance of the top 10 genera, for the same soil layer, was greater in GC than in BC, and the proportion was as smaller as less than 20%.

The fungal phyla were dominated by *Ascomycota* (GC5:64.17%, GC15:68.93%, GC25:64.82%, BC5:40.62%, BC15:41.36%, BC25:43.51%) and *Basidiomycota* (GC5:10.04%, GC15:9.90%, GC25:11.29%, BC5:12.26%, BC15:27.58%, BC25:33.90%) (Figure 4c). In the same soil layer, the relative abundances of *Ascomycota*, *Mortierellomycota*, and *Zoopagomycota* were higher in GC than in BC while the relative abundance of *Basidiomycota*, *Glomeromycota*, and *Chytridiomycota* were lower in GC than in BC. For each soil layer, the sum abundance of top 10 phyla was greater in GC than in BC. It indicated that BC reduced the relative abundance of fungal phyla, and this variation was larger than that of bacterial phyla, with a range of 50–90%.

The fungal genera were dominated by *unidentified Pyronemataceae* sp. (GC5:16.47%, GC15:31.39%, GC25:25.79%, BC5:7.65%, BC15:11.71%, BC25:16.09%), *Fusarium* (GC5:4.99%, GC15:9.15%, GC25:24.73%, BC5:5.17%, BC15:5.66%, BC25:8.06%), and *Solicoccozyma* (GC5:5.76%, GC15:6.77%, GC25:7.81%, BC5:3.51%, BC15:15.48%, BC25:24.75%) (Figure 4d). With the increase of soil depth, the abundance of *Fusarium* and *Solicoccozyma* increased while that of *unidentified Chaetomiaceae sp* decreased; the abundance of *unidentified Rozellomycota* sp. increased in GC and decreased in BC. In the same layer, the relative abundance of *unidentified Pyronemataceae* sp. and *unidentified Chaetothyriales* sp. were higher in GC than in BC while that of Phallus was lower in GC than in BC. The relative abundance of top 10 genera in the same soil layer was much greater in GC than in BC, accounting for 30–80%, which was higher than that of the bacterial genera. This suggested that BC had a significant effect on the regulation of fungal genera, which were mainly concentrated in the first 10 phyla.

### 3.4 Microbial variability analysis

Experimental treatments were subjected to analysis of variance at the phylum level (GC5–GC15, GC5–GC25, GC15–GC25, GC5–BC5, GC15–BC15, GC25–BC25, BC5–BC15, BC5–BC25, BC15–BC25). Species that differed significantly between treatments and had relative abundances greater than 0.1 were plotted (Table 2).

**Table 2:** Significance analysis of bacterial and fungal community phylum levels

	Group	Bacterial phylum	*P*	Group	Fungal phylum	*P*
GC	GC5-GC15	*Proteobacteria*	0.013*			
		*Acidobacteriota*	0.008**			
	GC5-GC25	*Proteobacteria*	0.036*			
		*Acidobacteriota*	0.008**			
GC-BC	GC5-BC5	*Proteobacteria*	0.006**	GC5-BC5	*Ascomycota*	0.012*
		*unidentified Bacteria*	0.049*			
		*Acidobacteriota*	0.011*	GC15-BC15	*Ascomycota*	0.009**
	GC15-BC15	*Proteobacteria*	0.001**		*Basidiomycota*	0.027*
	GC25-BC25	*unidentified Bacteria*	0.038*	GC25-BC25	*Basidiomycota*	0.009**
BC	BC5-BC25	*Proteobacteria*	0.005**	BC5-BC15	*Basidiomycota*	0.025*
	BC15-BC25	*Proteobacteria*	0.032*	BC5-BC25	*Basidiomycota*	0.010*

Note: Phyla with significant differences in bacterial and fungal communities in the same soil layer under different covering methods or in different soil layers with the same covering method. Missing values represent phylums with no significant differences.

*: *p* < 0.05; **: *p* < 0.01; ***: *p* < 0.001.

In the bacteria, the abundance of *Proteobacteria* differed the largest among treatments. As the soil depth increased, the difference of *Acidobacteriota* between BC and GC became unsignificant while that of *Proteobacteria* (*P* < 0.05) became highly significant (*P* < 0.01) (Table 2), indicating that BC has different regulatory effects on different bacteria. Three phyla were significantly different between GC5 and BC5, and one between GC15 and BC15 and between GC25 and BC25. At the genus level, a total of 35 genera from 6 phyla were significant different, mainly belonging to *Proteobacteria* (15 species) and *Acidobacteriota* (9 species).

In the fungi, *Ascomycota* and *Basidiomycota* were the greatest different phyla among these treatments. The fungal phylum was not different in each soil layer under GC. The abundance of *Basidiomycota* was significantly different in BC5–BC15 and BC5–BC25. It indicates that BC regulated the abundance of *Basidiomycota* to a significant level. as the depth gradually deepened, the significantly different phyla between GC and BC changed from *Ascomycota* to *Ascomycota* and *Basidiomycota*, and to *Basidiomycota* (Table 2). It suggested that BC regulated the fungal phyla depending on the soil depths. On the genus level, 24 differential genera were from *Ascomycota* (20 species), *Basidiomycota* (3 species), and *Rozellomycota* (1 species), respectively. It indicates that the significantly different species of fungi are mainly from *Ascomycota*.

### 3.5 Predictive analysis of soil microbial functions

**Figure 5:**
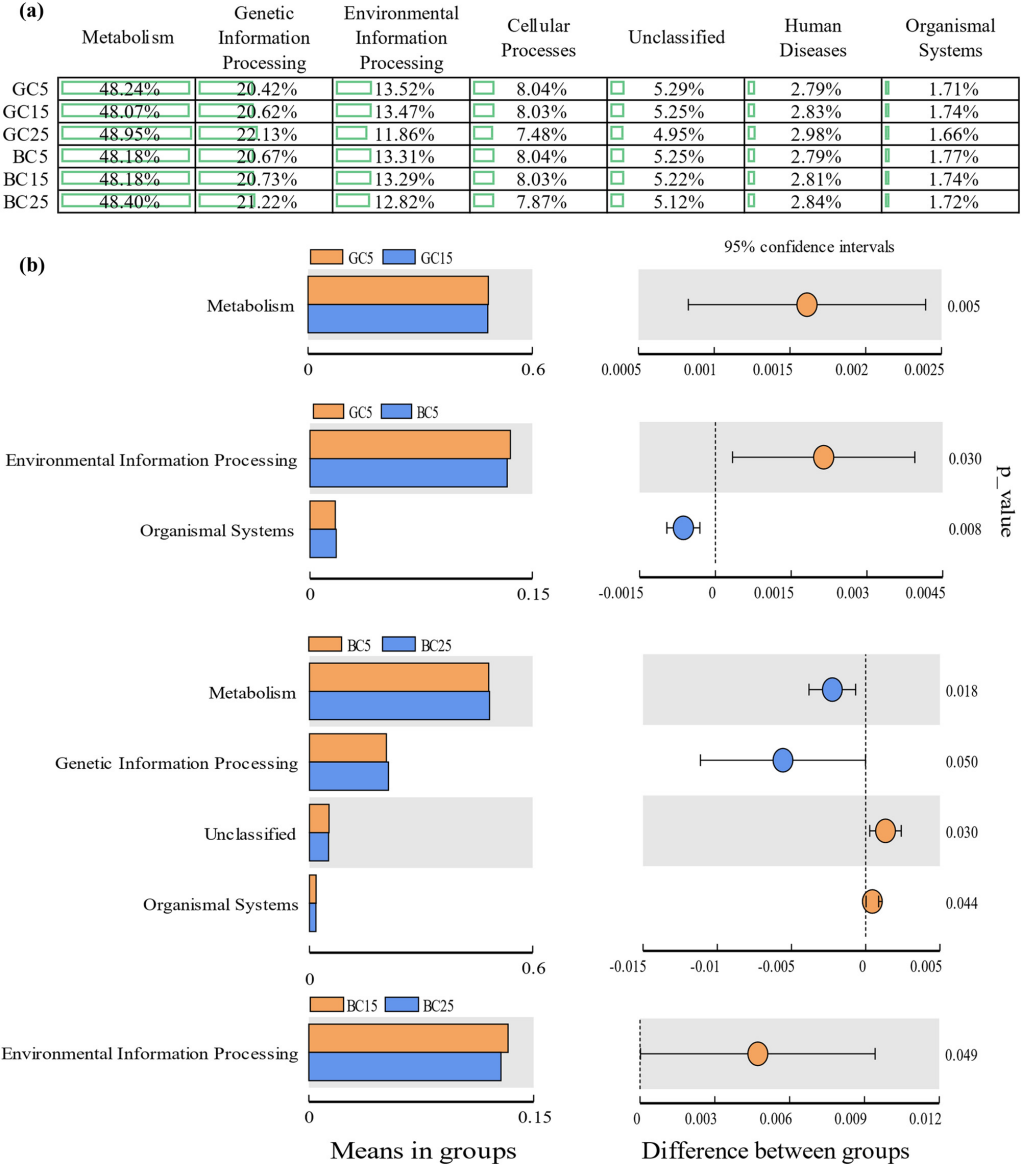
Bacterial function analysis (a: percentage of bacterial functions per treatment; b: bacterial function of significant differences between treatments at the same depth of soil or at different depths of soil in the same treatment).

The bacterial functions included metabolism, genetic information processing, and environmental information processing (Figure 5a). The differences in bacterial functions in different soil layers increased in BC when compared to GC, while the functional changes between GC and BC were smaller in the same soil layer (Figure 5b). This indicates that BC had a greater effect on the regulation of bacterial functions in the vertical direction than in the same soil layer. Bacterial functions for the same layer were most similar in the 15 cm depth BC and GC soil layers while they were most different in the 5 cm depth soil layer (Figure 7a). Indicating that bacterial functions in the 15 cm depth soil were the most stable.

**Figure 6:**
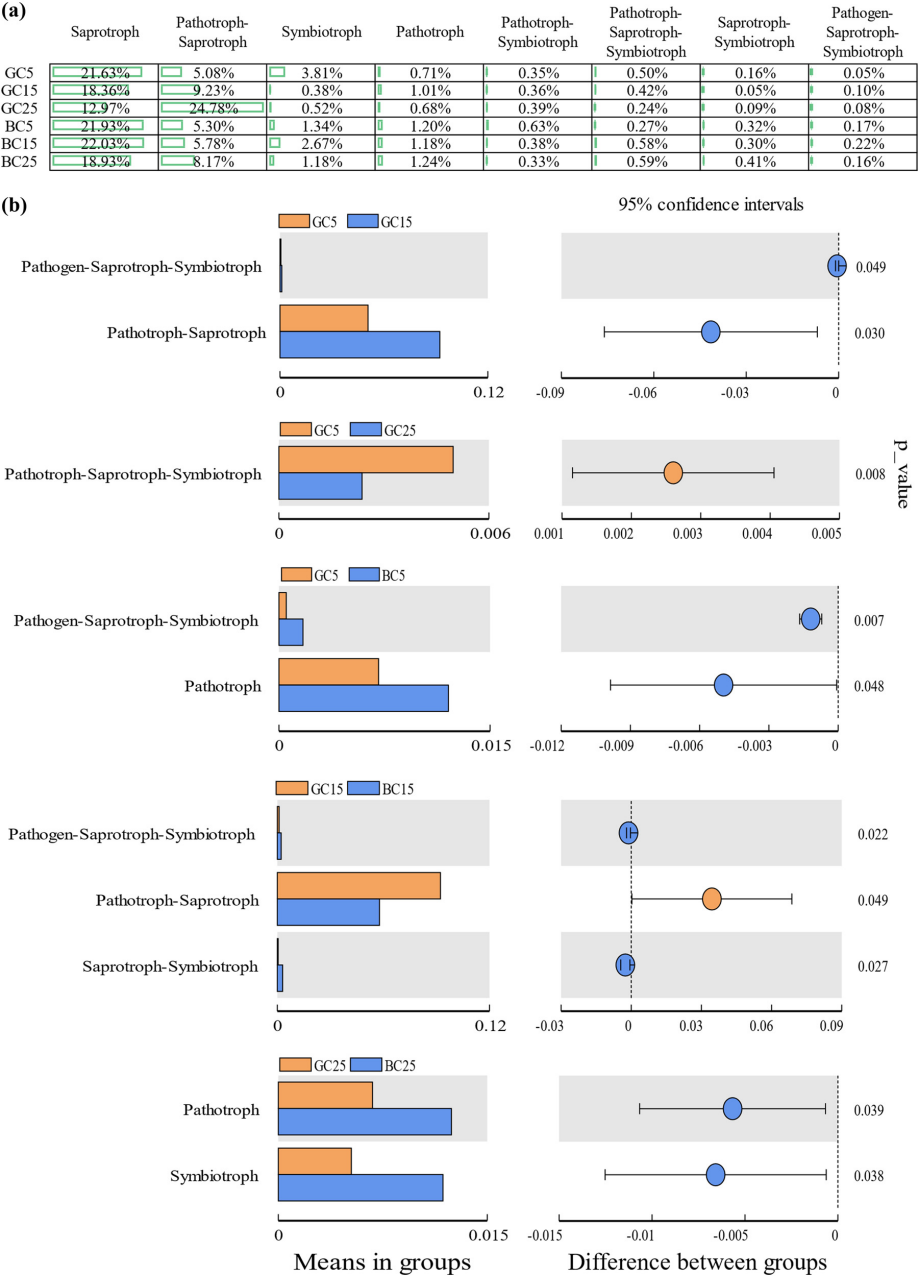
Fungal function analysis (a: percentage of fungal functions per treatment; b: fungal function of significant differences between treatments at the same depth of soil or at different depths of soil in the same treatment).

**Figure 7:**
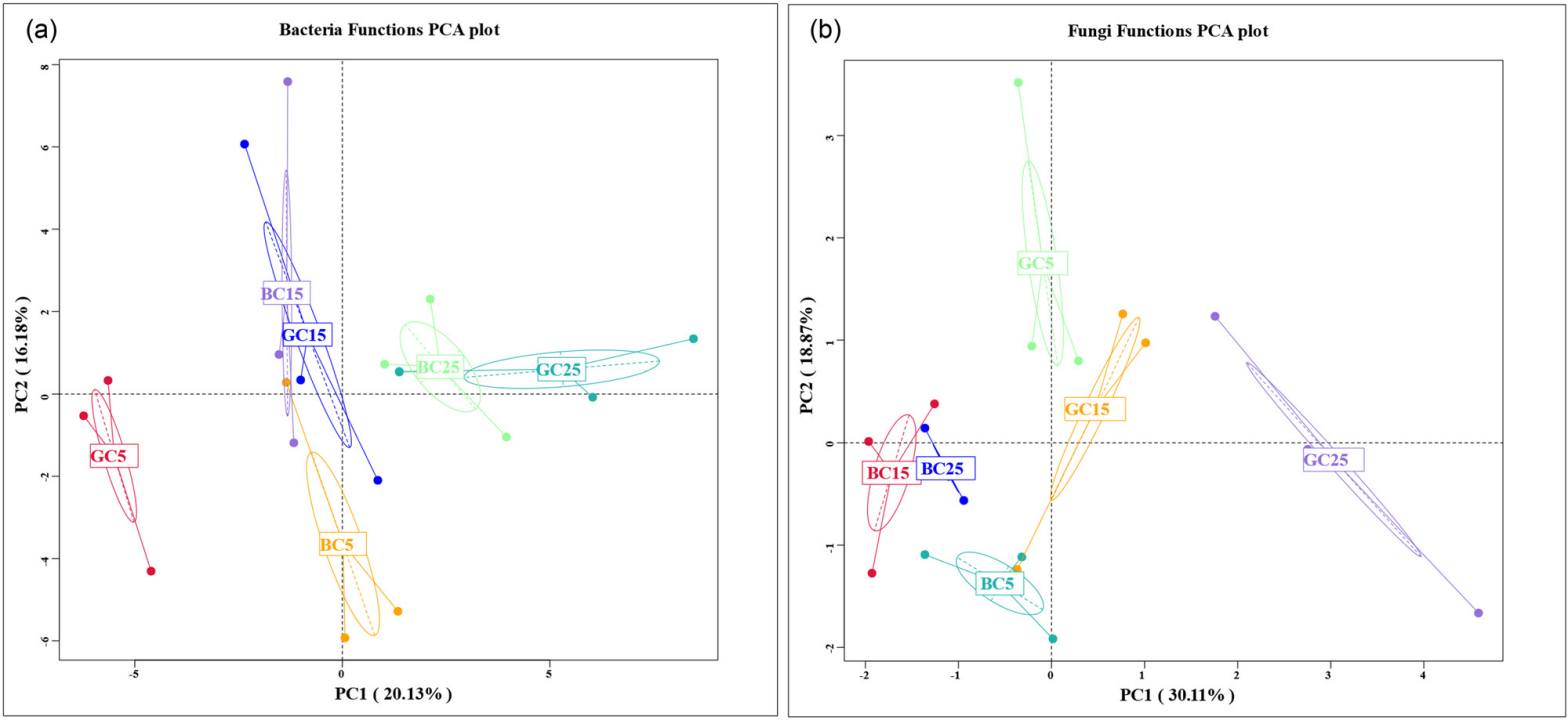
PCoA analysis of bacterial and fungal functions (a: PCOA diagram of bacteria functions; b: PCOA diagram of bacteria functions).

Fungal functions were dominated by Saprotroph and Pathotroph-Saprotroph. In the same soil layer, the relative abundance of Saprotroph was greater in BC than in GC (Figure 6a). There was no significant difference in function under BC. Fungal functions were significantly different between BC and GC in all the soil layers (Figure 6b). The fungal functions were the most similar in the 15 cm depth soil layer while the most different in 25 cm depth soil layer; the variability of fungal functions among soil layers was greater in GC than in BC. BC had a larger regulating effect on fungal function than on bacterial function (Figure 7b).

### 3.6 Correlation analysis between soil chemical properties and soil microorganisms

**Figure 8:**
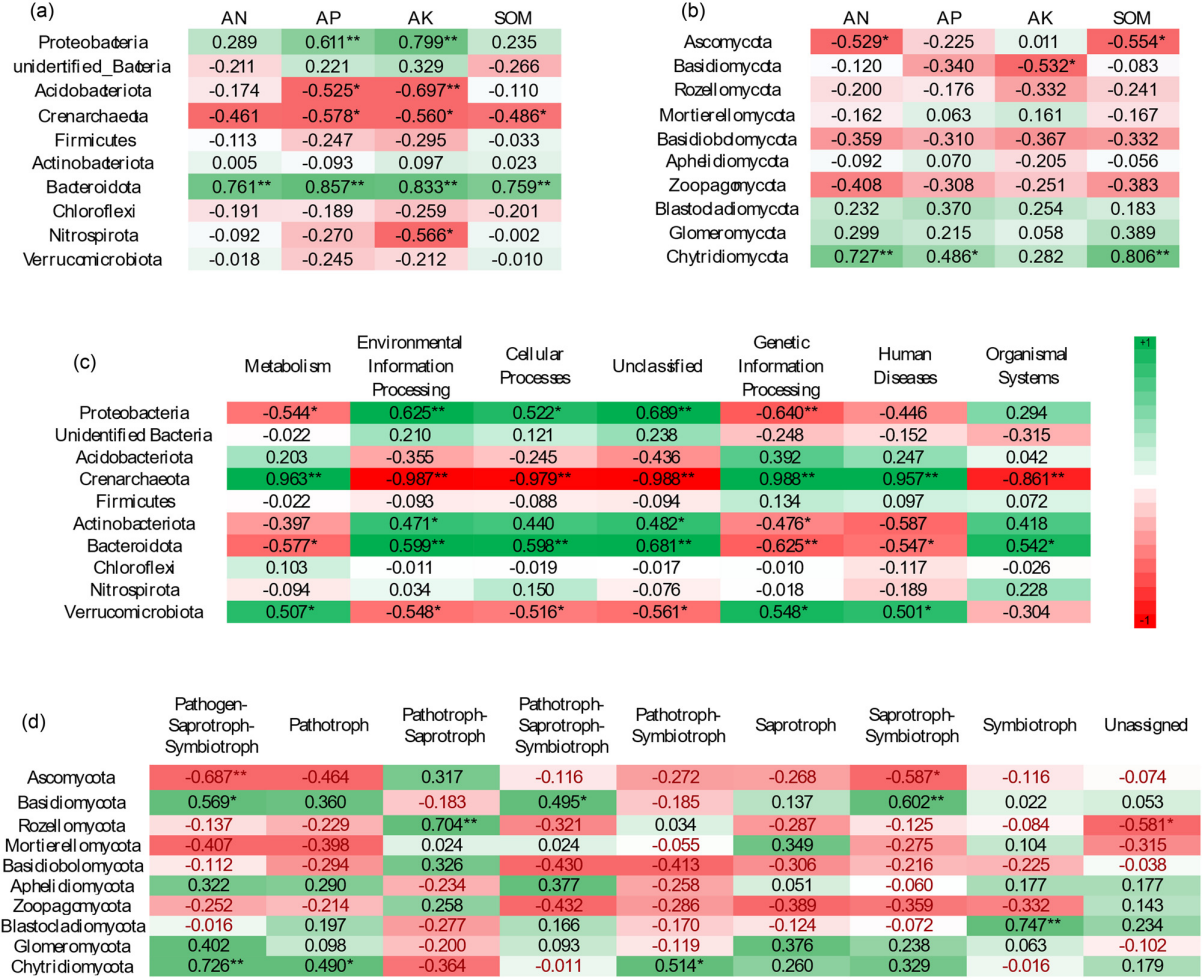
Correlation analysis between soil chemical properties and soil microorganism (a: correlation analysis between bacterial phyla and soil properties; b: correlation analysis between fungal phyla and soil properties; c: correlation analysis of bacterial phyla and bacterial functions; d: correlation analysis of fungal phyla and fungal functions).

**Figure 9:**
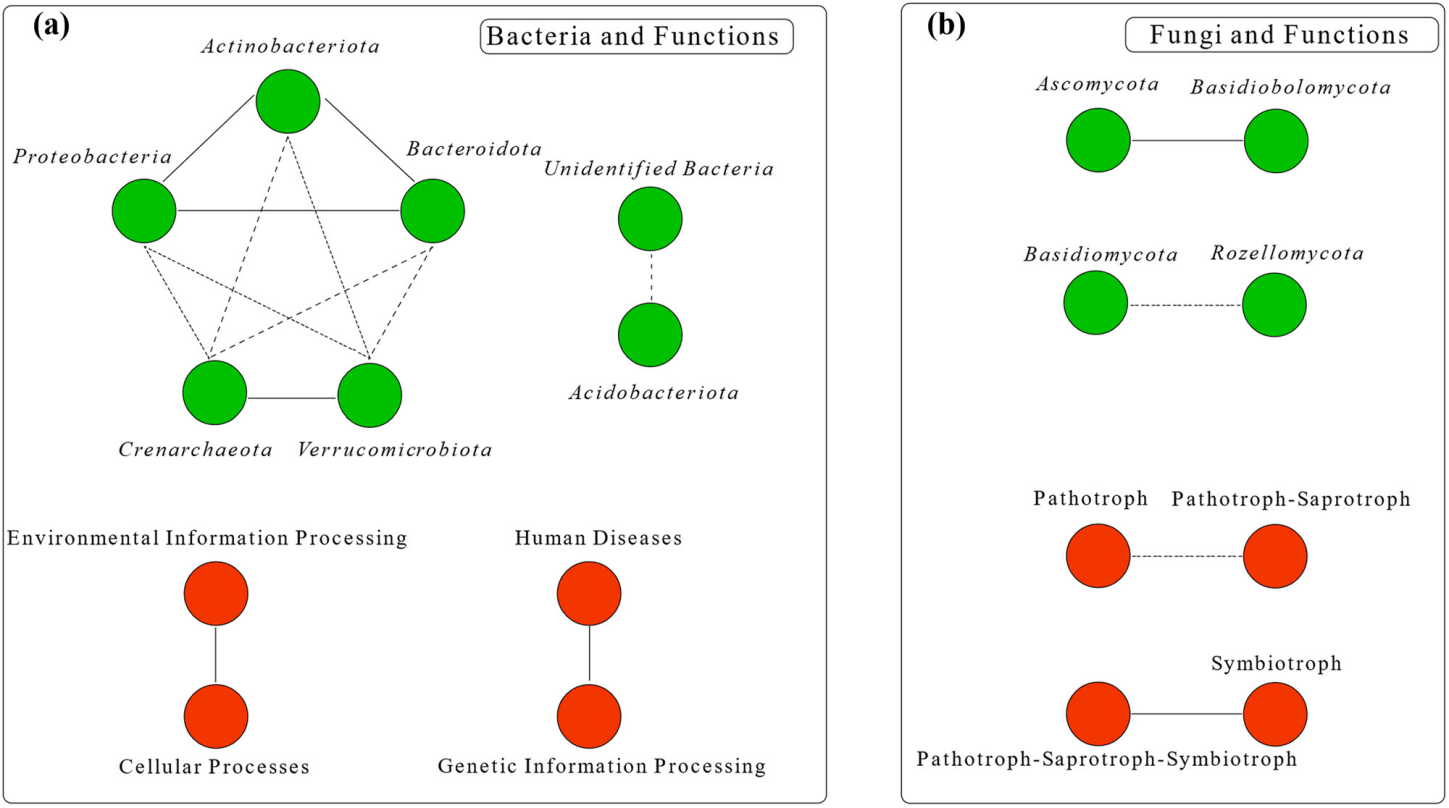
Analysis of relationships between microorganisms (functions) (two randomly selected microorganisms (functions) and whether their correlations with all functions (microorganisms) are consistent or opposite, with the solid line representing consistent relationships and the dashed line representing opposite relationships. Solid lines between different microorganisms or functions indicated synergy effects while dashed lines indicated antagonisms. a: Relationships of bacteria and functions; b: Relationships of fungi and functions).

Both AP and AK were strongly correlated with the abundances of *Proteobacteria*, *Acidobacteriota*, *Crenarchaeota*, and *Bacteroidota* (*P* < 0.05), while AN and SOM were strongly correlated with the abundance of *Bacteroidota* (*P* < 0.01). This indicates that AP and AK correlated more strongly with bacterial phylum than AN and SOM did. *Bacteroidota* correlated extremely with soil chemical properties (*P* < 0.01, Figure 8a). The abundance of *Ascomycota* negatively correlated with AN and SOM (*P* < 0.05) and the abundance of *Basidiomycota* negatively correlated with AK (*P* < 0.05), while the abundance of *Chytridiomycota* positively correlated with AN (*P* < 0.01), AP (*P* < 0.05), and SOM (*P* < 0.01) (Figure 8b). In summary, bacteria had a stronger correlation with soil chemical properties than fungi (Figure 9).

*Proteobacteria* (except human diseases, organismal systems), *Verrucomicrobiota* (except organismal systems) *Bacteroidota*, and especially *Crenarchaeota*, were significantly correlated with bacterial functions. We inferred that *Crenarchaeota* and *Bacteroidota* are mainly involved in the composition of bacterial function (Figure 8c). Compared to bacteria, the first 10 phyla of fungi were weakly correlated with fungal functions. *Basidiomycota* and *Chytridiomycota* were significantly correlated with three fungal functions (*P* < 0.05), *Ascomycota* and *Rozellomycota* were significantly correlated with two fungal functions (*P* < 0.05), and *Blastocladiomycote* was highly significantly correlated with one fungal function (*P* < 0.01) (Figure 8d).

It shows that the relationship between bacteria and bacteria is more complex than that between fungi and fungi.

## 4 Discussion

Crops covering is strongly associated with soil microorganisms [36]. Soil nutrients were increased to different degrees in BC. This is similar to that straw return increased soil N and organic matter content [37,38] and that raw grass can substantially nourish the soil by effectively improving the physical and chemical properties of orchard soil and increasing the content of organic matter [39]. In the study, the effect of BC gradually diminished in both directions of SOM-AN-AP-AK and soil depth. If the *F* value of GC > BC is defined as negative, it can be found that *F*(SOM) > *F*(AN) > *F*(AP) > *F*(AK) in the Coverage model, i.e., the sensitivity of soil chemical properties to the environment should be SOM > AN > AP > AK. It seemed that the effect of BC on the soil shows infiltration effect. The response of SOM and AN to BC is faster, while the response of AP and AK is slower. Over time, the effect of BC on the soil will become larger and gradually exceed the soil depth, the soil chemical properties keep improving, and it is possible that *F* (AK, BC) > *F* (AK, GC). This is consistent with Yan’s results that organic matter, ammoniacal nitrogen, and AP in the branch-covered soil for 3 years were greater than that in the control, while AK was less than the control [40].

Crops-covering can increase the amount and diversity of microbial communities in the soil [41,42]. Microbial abundance, such as pathogens, was reduced by planting crops on grapes [23]. In the research: it was found that BC increased microbial diversity and homogeneity, increased bacterial abundance, and decreased fungal abundance. Among them, the 15 cm soil layer was highly aggregated and stable. It might that the depth of 15 cm soil layer was suitable; a deeper the soil depth with a stronger the dispersion; and the shallower soil was easily influenced by the surface environment. In NMDS analysis, GC25 had the strongest dispersion both in bacteria and fungi and was much diminished in BC; while GC5 and GC15 were more intensive and changed less compared to BC5 and BC15; and BC15 dispersion was enhanced compared to the very dense GC15. It indicates that BC has a moderating effect on species community richness, and there is a certain threshold of the moderating effect, which increases the difference when the community difference is too small and decreases the difference if the difference is too large. The box plots were consistent with the results of NMDS analysis: both bacteria and fungi were denser in soil layer 15; the more dispersed GC25 was significantly improved compared to BC25; the denser GC5 and GC15 were slightly adjusted. This further suggests the possibility of a threshold for BC regulation.

It showed that the bacterial community mainly includes *Proteobacteria* and *Acidobacteriota* [43], which was similar to the present results. The relative abundance of the top 10 phyla was similar in each treatment. In addition, we also found that: BC had a certain reduction effect on *Proteobacteria*, etc., which accounted for a higher percentage, and may have an inhibitory effect on diseases such as Pear Fire Blight caused by *Proteobacteria*; BC had a positive effect on improving the abundance of *Acidobacteriota* and *Firmicutes*, etc., which had a lower relative abundance. It indicated that BC had different improvement effects on bacterial phyla with different abundances, and it was consistent with NMDS analysis. Among the bacterial genera, the relative total abundance of the top 10 genera of GC was greater than that of BC. Consistent with the bacterial phylum, BC also regulated the genera of bacteria in different ways and at different degrees. It is worth mentioning that the top 10 genera of bacteria only accounted for 8.89–16.92% of the total genera, which is much lower than the percentage of bacterial phylum, indicating that bacterial genera are more evenly distributed. Similar to bacteria, the regulatory effects of BC on different species of fungi also differed, with a decreasing effect on the more abundant *Ascomycota* and *unidentified Pyronemataceae* sp. and an increasing effect on the less abundant of *Basidiomycota* and *Solicoccozyma*. Differently, BC reduced the fungal abundance and the abundance of the first 10 phyla changed more largely. This indicates that fungi are more sensitive to the environment, which is consistent with the findings that fungi are more sensitive than bacteria to the type of weeds and tillage [44,45]. It is well known that most of the pathogens such as Ring Rot Disease, Canker, and Brown Spot Diseases belong to the *Ascomycota*. Therefore, can crops-covering reduce the pathogenic bacterial base by reducing the *Ascomycota*, to adjust the microbial community structure and then reduce the occurrence of pests and diseases?

The microbial function analysis showed that the relative abundance of bacterial functions in the same soil layer in GC was much different from those in BC, except for those between GC5 and BC5 with a significant difference in the abundance of fungal functions in the same soil layer differed greatly and all three soil layers produced significant difference functions. The number of bacterial functions with significant differences among different soil layers increased in BC compared with GC, while the opposite went for the fungal functions. Possible reasons could be bacterial functions of three soil layers in BC showed dispersed changes while fungal functions showed aggregated changes, which further indicated that BC increased the bacterial functional diversity and decreased the fungal functional diversity. This is inconsistent with the previous study that BC increased bacterial and fungal diversity, and the reason might be that the increased fungal species in BC were mostly with the same functions, thus leading to a decrease in the fungal functional diversity while increasing the fungal diversity. The stronger sensitivity of fungi than bacteria may be due to the smaller number of fungi; and the increasing amount of Saprotroph may help improve the soil and the apparent decreasing in Pathotroph-Saprotroph may help reduce the occurrence of pests and diseases.

Soil properties contribute largely to the soil microbial community structure [46]. In the correlation analysis, AP and AK were more strongly correlated with bacterial phyla and less correlated with fungal phyla, indicating that AP and AK were more closely related to bacterial community. *Crenarchaeota* was highly correlated with bacterial functions; *Bacteroidota* was highly correlated with soil chemical properties and bacterial functions. It is inferred that *Crenarchaeota* and *Bacteroidota* played a key role in bacterial function. The top 10 fungal phyla were weakly correlated with fungal functions, and the phyla determining fungal functions might be mainly in phyla with relatively low abundance.

In this article, species with consistently increasing or decreasing relative abundance as the soil depth increases were properly discussed, while the microorganisms with small changes or inconsistent trends are not highlighted, which requires further evaluation by adding soil depth treatments. Further studies are also needed to clarify the threshold of microbial regulation by BC.

## 5 Conclusion

Soil chemical properties were strongly correlated with bacterial phyla and their functions but weakly correlated with fungal phyla and their functions. Preliminarily, it was demonstrated that there was a threshold value for the adjustment of microbial community structure by BC, and the stability and consistency of microorganisms were better in 15–25 cm soil layers. BC enhanced soil chemical properties, increased bacterial abundance, reduced fungal abundance, increased microbial diversity, and regulated species structure and microbial functions. BC can be used as an alternative technique to GC.
